# Supplementary figures and images for: Homeostatic control of stearoyl desaturase expression via patched-like receptor PTR-23 ensures the survival of C. elegans during heat stress
Source: PLoS Genet. 2023 Dec 18;19(12):e1011067. doi: 10.1371/journal.pgen.1011067 (PMC10727360; doi:10.1371/journal.pgen.1011067)

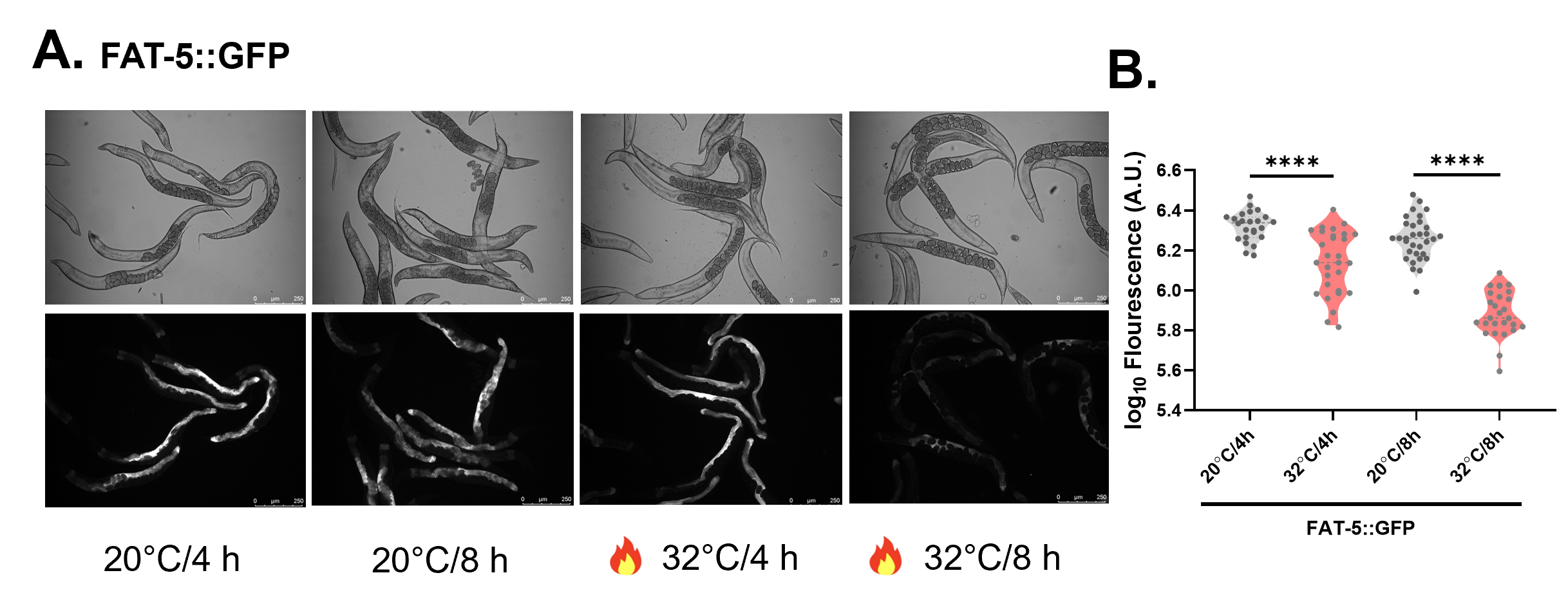

Supplement: S1 Fig — (A) FAT-5::GFP expression in ctrl and HS animals at indicated times and temperatures (scale bar, 250 μm) and their (B) quantification. Statistical significance for (B) was calculated using a post-hoc Dunnett test. Ns (not significant), * P ≤ 0.05; ** P ≤ 0.01; *** P ≤ 0.001; **** P ≤ 0.0001. (TIF) [file pgen.1011067.s001.tif]

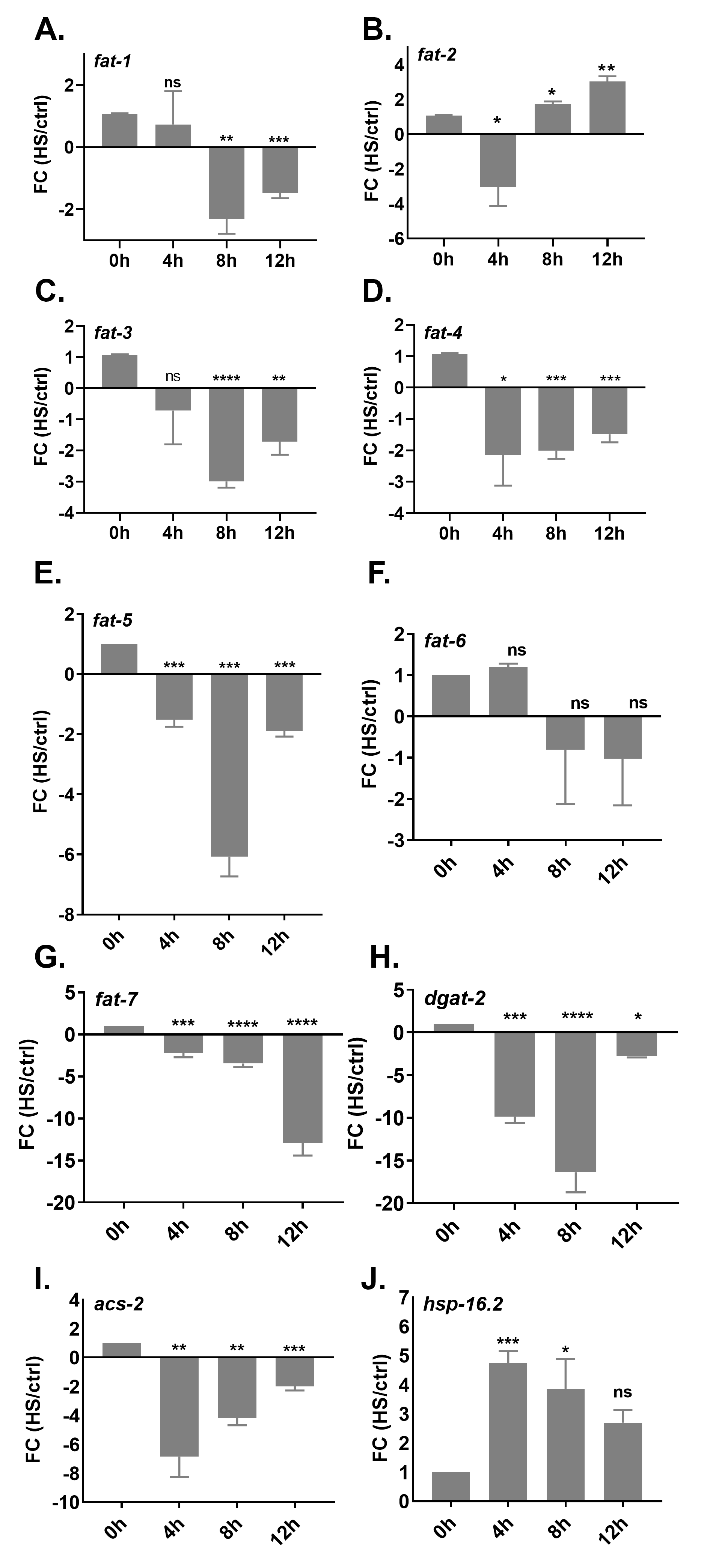

Supplement: S2 Fig — qPCR analysis of (A) fat-1, (B) fat-2, (C) fat-3, (D) fat-4, (E) fat-5, (F) fat-6, (G) fat-7, (H) dgat-2, (I) acs-2, and (J) hsp-16.2 in HS (32°C) over ctrl (20°C) WT animals. ns, non-significant; *P < 0.05; **, P < 0.01; ***, P < 0.001; ****, P < 0.0001 as determined by unpaired ‘t’ test with Welch’s correction. Error bars represent SEM. (TIF) [file pgen.1011067.s002.tif]

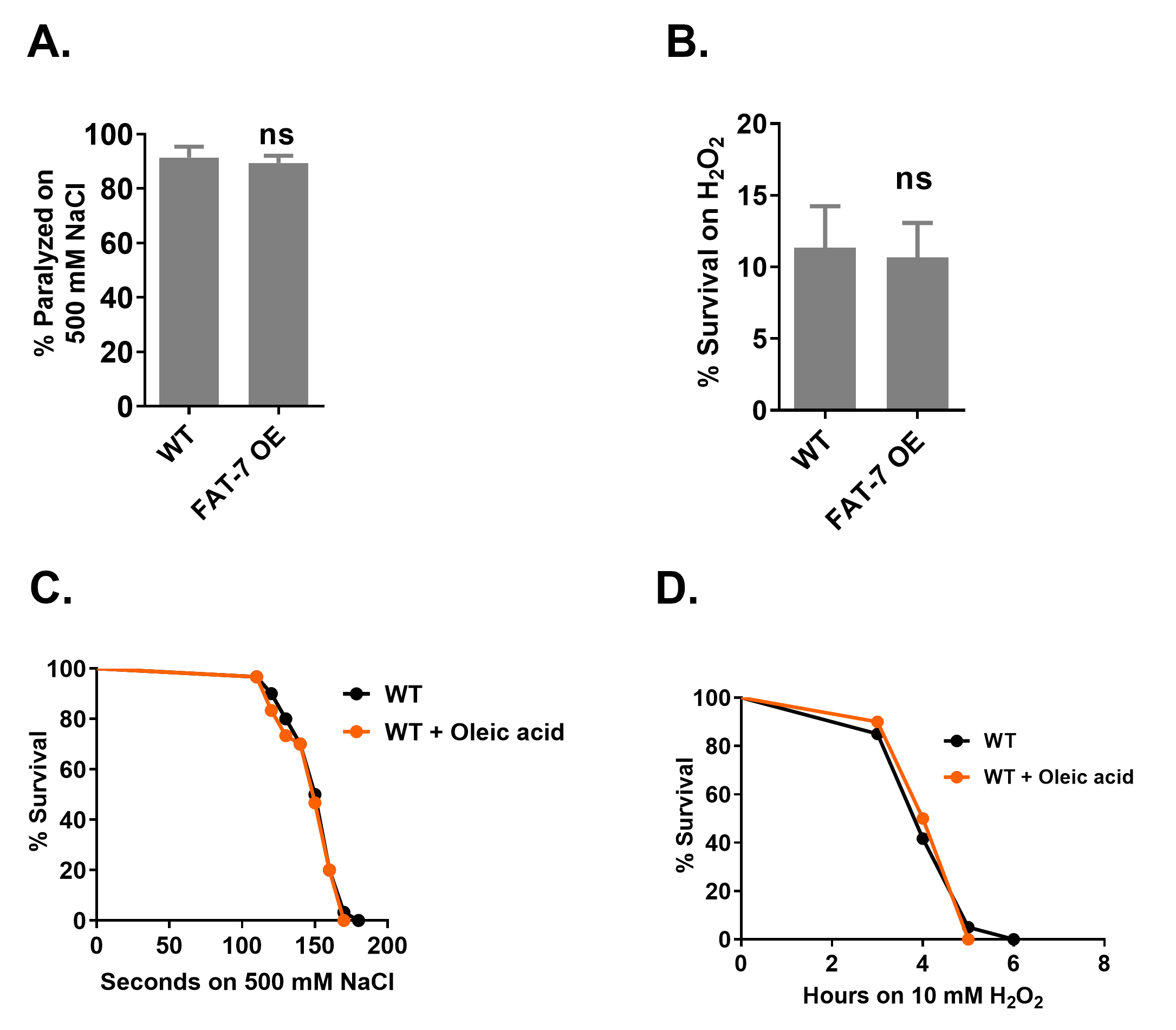

Supplement: S3 Fig — (A) Survival of WT and FAT-7 OE animals during osmotic stress of 500 mM NaCl. (B) Survival of WT and FAT-7 OE animals during acute oxidative stress upon 6-hour exposure to 10 mM H2O2. (C) Kaplan-Meier survival curves of WT and WT supplemented with oleic acid during osmotic stress upon exposure to 500 mM NaCl (P = 0.7329; N = 25–30; n = 3). (D) Kaplan-Meier survival curves of WT and WT supplemented with oleic acid during oxidative stress upon exposure to 10 mM H2O2 (P = 0.6384; N = 25–30; n = 3). ns (not significant).as determined by unpaired Student’s ‘t’ test. Error bars represent SEM. (TIF) [file pgen.1011067.s003.tif]

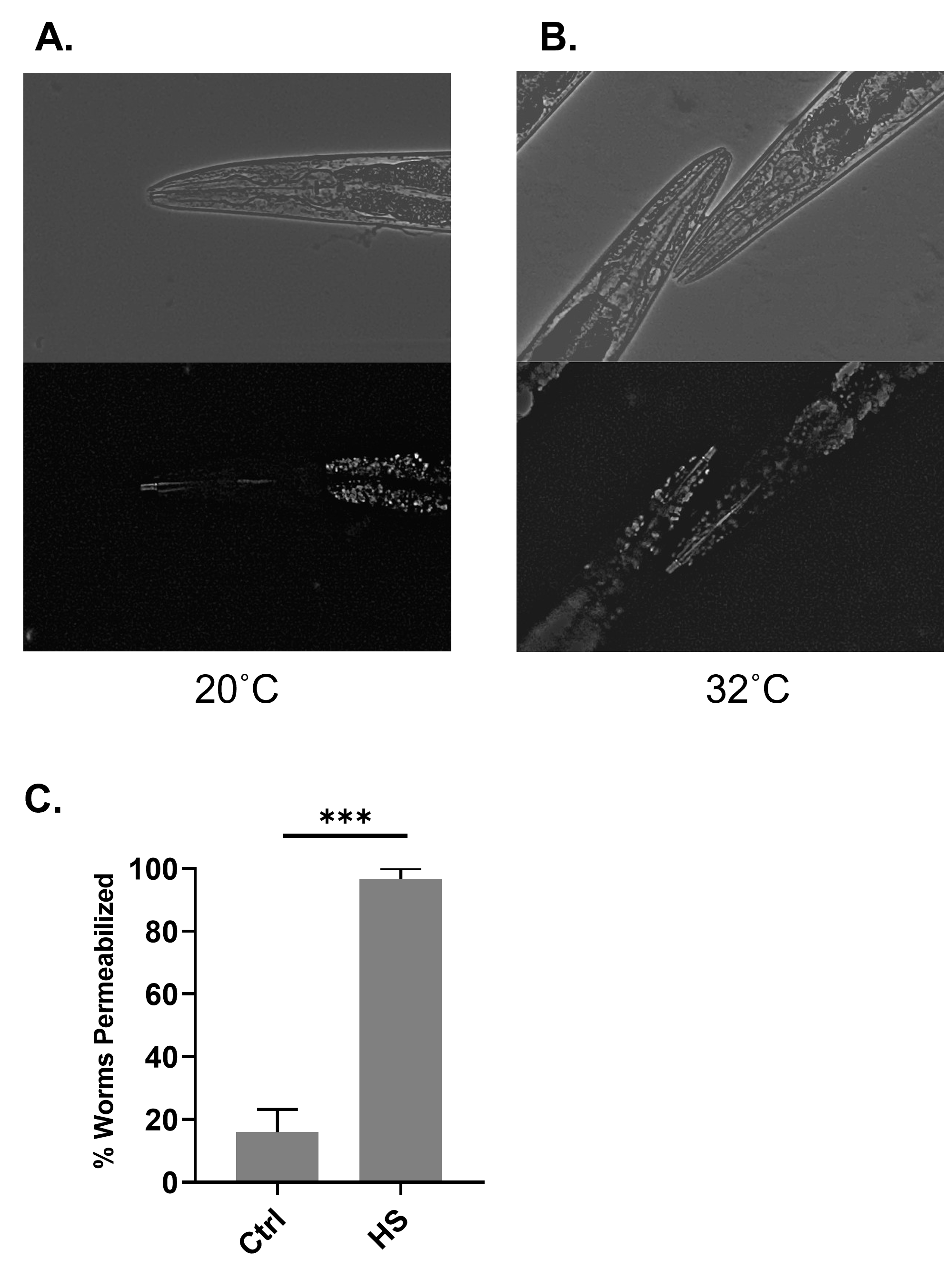

Supplement: S4 Fig — DIC and DAPI filter images of WT adults grown for 24 h at (A) 20°C or (B) 32°C followed by staining with Hoechst stain. (C) Percentage of worms permeabilized as quantified by Hoechst staining in the region of interrest (ROI) around the pharynx. ***, P < 0.001 as determined by unpaired ‘t’ test with Welch’s correction. Error bars represent SEM. (TIF) [file pgen.1011067.s004.tif]

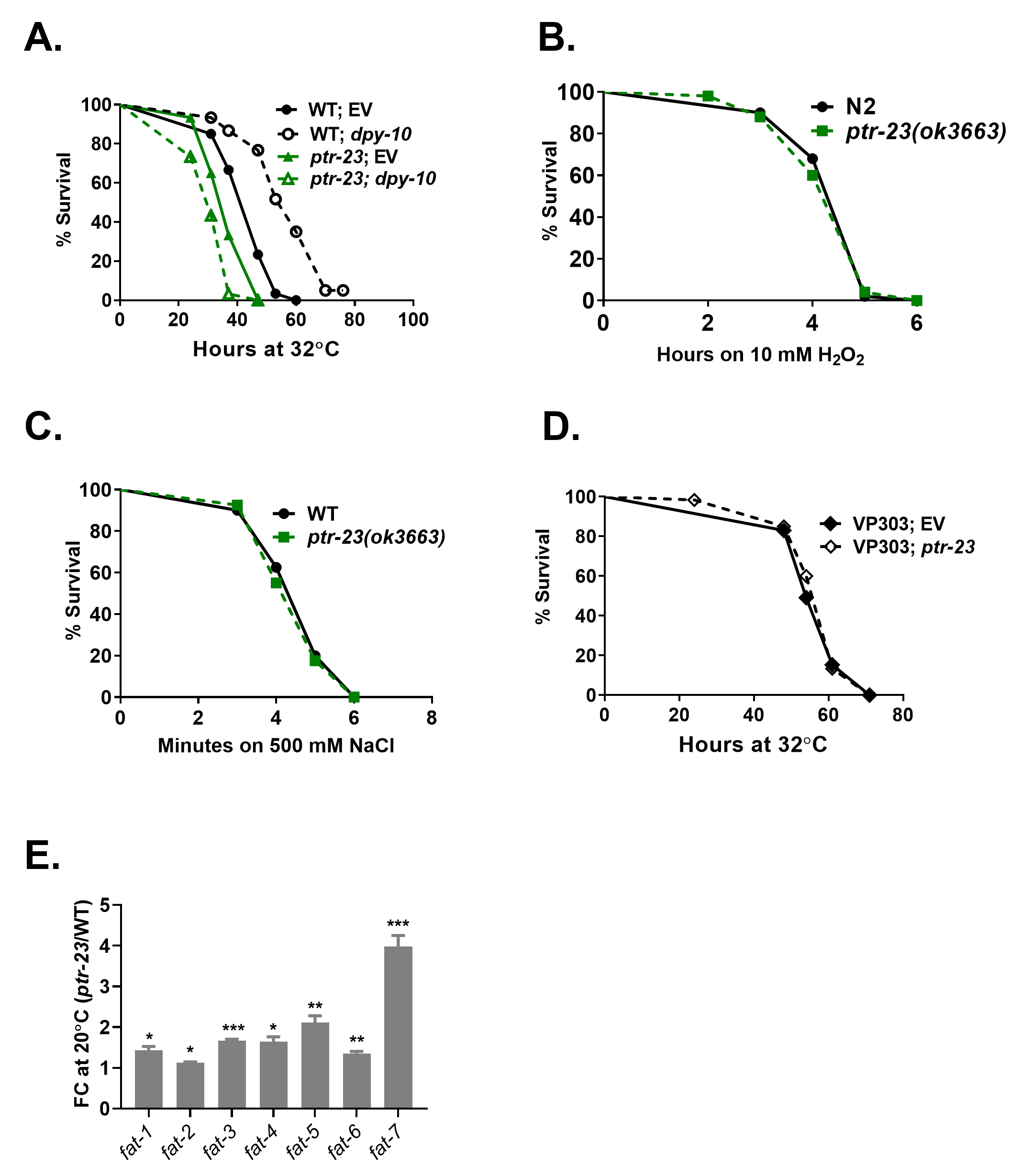

Supplement: S5 Fig — (A) Kaplan Meier survival curves of WT and ptr-23 animal with dpy-10 RNAi at 32°C (P<0.0001; N = 25-3-; n = 3). Kaplan-Meier survival curves of N2 and ptr-23(ok3663) animals during (C) oxidative stress (P = 0.5671; N = 25–30; n = 3) and (D) osmotic stress (P = 0.6442; N = 25–30; n = 3). (D) Kaplan Meier survival curves of VP303, intestinal RNAI animals with ptr-23 RNAi (P = 0.5906; N = 25–30; n = 3). (E) Transcript levels of fatty acid desaturase genes in ptr-23(ok3663) mutants compared to WT animals. *P < 0.05; **, P < 0.01; ***, P < 0.001 as determined by unpaired ‘t’ test with Welch’s correction. Error bars represent SEM. (TIF) [file pgen.1011067.s005.tif]
