# Supplementary material for: Homeostatic control of stearoyl desaturase expression via patched-like receptor PTR-23 ensures the survival of C. elegans during heat stress
Source: PLoS Genet. 2023 Dec 18;19(12):e1011067. doi: 10.1371/journal.pgen.1011067 (PMC10727360; doi:10.1371/journal.pgen.1011067)
Supplement: S1 Table — (DOCX) [file pgen.1011067.s006.docx]

Table S1. Fatty acid analysis by Gas chromatography-mass spectrometry

| **Control worms (20°C/8h)** | **WT (n=12)** | | ***dpy-10 (vs2003)***  **(n=9)** | | ***ptr-23* (n=5)** | | ***dpy-10 (vs2003); ptr- 23* (n=3)** | |
| --- | --- | --- | --- | --- | --- | --- | --- | --- |
| **Fatty acid (% of total)** |  | |  | |  | |  | |
| **14:0** | 0.59 ± 0.02 | | 0.79 ± 0.05  (**) | | 0.62 ± 0.03  (ns) | | 0.8 ± 0.03  (**) | |
| **15iso** | 2.58 ± 0.06 | | 2.64 ± 0.24  (ns) | | 2.37 ± 0.17  (ns) | | 1.4 ± 0.03  (****) | |
| **16:0** | 2.93 ± 0.07 | | 4.7 ± 0.35  (****) | | 3.21 ± 0.12  (ns) | | 5.7 ± 0.02  (****) | |
| **17iso** | 3.8 ± 0.07 | | 3.33 ± 0.07  (**) | | 3.54 ± 0.07  (ns) | | 2.46 ± 0.02  (****) | |
| **16:1** | 0.89 ± 0.03 | | 1.45 ± 0.06  (**) | | 1.05 ± 0.07  (*) | | 2.5 ± 0.06  (****) | |
| **17d** | 10.84 ± 0.47 | | 10.1 ± 0.19  (ns) | | 11.59 ± 1.01  (ns) | | 15.78 ± 0.73  (***) | |
| **18:0** | 5.25 ± 0.12 | | 5.61 ± 0.11  (ns) | | 5.38 ± 0.17  (ns) | | 5.55 ± 0.06  (ns) | |
| **18:1n-9** | 2.05 ± 0.11 | | 1.44 ± 0.04  (***) | | 2.34 ± 0.07  (ns) | | 2.06 ± 0.05  (ns) | |
| **18:1n-7** | 17.02 ± 0.17 | | 17.8 ± 0.27  (*) | | 16.92 ± 0.17  (ns) | | 22.22 ± 0.43  (****) | |
| **18:2** | 6.54 ± 0.08 | | 5.06 ± 0.12  (****) | | 6.32 ± 0.15  (ns) | | 5.41 ± 0.11  (****) | |
| **19d** | 11.6 ± 0.31 | | 10.6 ± 0.22  (ns) | | 11.43 ± 0.35  (ns) | | 11.65 ± 0.2  (ns) | |
| **18:3** | 1.63 ± 0.04 | | 1.57 ± 0.05  (ns) | | 1.9 ± 0.05  (**) | | 1.37 ± 0.05  (*) | |
| **20:3** | 3.39 ± 0.12 | | 4.21 ± 0.12  (***) | | 3.94 ± 0.11  (*) | | 3.1 ± 0.15  (ns) | |
| **20:4** | 1.47 ± 0.04 | | 3.15 ± 0.05  (****) | | 1.59 ± 0.05  (ns) | | 1.83 ± 0.13 (**) | |
| **20:4n-3** | 5.07 ± 0.1 | | 4.66 ± 0.14  (*) | | 4.83 ± 0.15  (ns) | | 3.19 ± 0.04  (****) | |
| **20:5** | 20.2 ± 0.76 | | 17.1 ± 0.3  (**) | | 18.26 ± 0.73  (ns) | | 11.72 ± 0.4  (****) | |
| **Heat stressed**  **worms (32°C/8h)** | **WT (n=12)** | | ***dpy-10 (vs2003)***  **(n=12)** | | **ptr-23 (n=5)** | | ***dpy-10 (vs2003); ptr- 23* (n=3)** | |
| **Fatty acid (% of total)** |  | |  | |  | |  | |
| **14:0** | 0.94 ± 0.04 | (****) | 0.81 ± 0.02  (*) | (ns) | 0.93 ± 0.03  (ns) | (***) | 0.91 ± 0.04  (ns) | (ns) |
| **15iso** | 3.44 ± 0.12  (****) | | 3.14 ± 0.27  (**) | (ns) | 3.4 ± 0.15  (ns) | (**) | 1.27 ± 0.03  (****) | (*) |
| **16:0** | 4.54 ± 0.22  (****) | | 5.97 ± 0.16  (****) | (**) | 4.25 ± 0.21  (ns) | (**) | 7.37 ± 0.39  (****) | (*) |
| **17iso** | 3.99 ± 0.12 | (ns) | 3.53 ± 0.02  (****) | (ns) | 4.13 ± 0.1  (ns) | (**) | 2.27 ± 0.05  (****) | (ns) |
| **16:1** | 0.82 ± 0.03 | (ns) | 1.03 ± 0.02  (*) | (****) | 1.02 ± 0.05  (**) | (ns) | 2.0 ± 0.05  (****) | (**) |
| **17d** | 9.8 ± 0.86 | (ns) | 9.7 ± 0.24  (ns) | (ns) | 12.33 ± 0.7  (ns) | (ns) | 15.65 ± 0.79  (**) (ns) | |
| **18:0** | 5.47 ± 0.33 | | 6.19 ± 0.14 | | 5.22 ± 0.26 | | 5.75 ± 0.21 | |

|  | (ns) | (ns) (**) | (ns) (ns) | (ns) (ns) |
| --- | --- | --- | --- | --- |
| **18:1n-9** | 2.33 ± 0.15  (ns) | 1.66 ± 0.04  (**) (**) | 2.54 ± 0.07  (ns) (ns) | 1.9 ± 0.06  (ns) (ns) |
| **18:1n-7** | 15.91 ± 0.18  (***) | 18.18 ± 0.2  (****) (ns) | 16.45 ± 0.08  (ns) (ns) | 21.43 ± 0.22  (****) (ns) |
| **18:2** | 11.71 ± 0.48  (****) | 7.01 ± 0.1  (****) (****) | 10.4 ± 0.22  (ns) (****) | 7.16 ± 0.23  (***) (**) |
| **19d** | 9.95 ± 0.48  (ns) | 9.78 ± 0.14  (ns) (*) | 11.24 ± 0.3  (ns) (ns) | 11.41 ± 0.46  (ns) (ns) |
| **18:3** | 0.8 ± 0.06  (****) | 0.59 ± 0.03  (ns) (****) | 0.68 ± 0.03  (ns) (****) | 0.52 ± 0.06  (ns) (***) |
| **20:3** | 2.05 ± 0.11  (****) | 3.97 ± 0.6  (****) (ns) | 2.53 ± 0.08  (*) (****) | 3.02 ± 0.03  (**) (ns) |
| **20:4** | 1.16 ± 0.08  (**) | 2.86 ± 0.03  (****) (***) | 1.01 ± 0.03  (ns) (****) | 1.57 ± 0.07  (*) (ns) |
| **20:4n-3** | 4.18 ± 0.13  (****) | 4.31 ± 0.07  (*) (ns) | 4.22 ± 0.14  (ns) (*) | 3.13 ± 0.09  (**) (ns) |
| **20:5** | 16.56 ± 0.88  (**) | 14.94 ± 0.18  (ns) (****) | 14.17 ± 0.31  (ns) (***) | 10.71 ± 0.5  (**) (ns) |

Significance obtained by Student’s *t*-test

* against wild type WT at the same temperature (across rows)

*dpy-10* over WT

*ptr-23* over WT

*dpy-10;ptr-23* over WT

*dpy-10* HS over WT HS

*ptr-23* HS over WT HS

*dpy-10;ptr-23* HS over WT HS

***** HS worms against the control of same genetic background (across columns)

N2 HS vs N2 ctrl

*dpy-10* HS over *dpy-10* ctrl

*ptr-23* HS over *ptr-23* ctrl

*dpy-10;ptr-23* HS over *dpy-10;ptr-23* ctrl
