# Supplementary material for: Homeostatic control of stearoyl desaturase expression via patched-like receptor PTR-23 ensures the survival of C. elegans during heat stress
Source: PLoS Genet. 2023 Dec 18;19(12):e1011067. doi: 10.1371/journal.pgen.1011067 (PMC10727360; doi:10.1371/journal.pgen.1011067)
Supplement: S2 Table — (DOCX) [file pgen.1011067.s007.docx]

**Table S2. Statistics for Survival assays.**

| **Condition** | **Genotype** | **Number of animals (Assayed/Censored)** | **TD_50_ in Hours** | **p-value** |
| --- | --- | --- | --- | --- |
| **Heat Stress Survival @ 32˚C** | **WT**  **FAT-7::GFP OE** | **69/0**  **69/0** | **41.16**  **34.27** | **<0.0001** |
|  | **WT**  **FAT-7::GFP OE** | **72/0**  **69/0** | **40**  **34.76** | **0.0005** |
|  | **WT**  **FAT-7::GFP OE** | **65/0**  **64/0** | **41.07**  **34.84** | **0.0002** |
|  | **WT**  **FAT-7::GFP OE** | **62/0**  **70/0** | **40.4**  **34.13** | **0.0002** |
| **Heat Stress Survival @ 32˚C**  **Heat Stress Survival @ 32˚C** | **WT**  ***dpy-10***  **FAT-7::GFP OE**  ***dpy-10* ; FAT-7::GFP OE**  ***dpy-10* ; *ptr-23* ; FAT-7::GFP OE** | **54/9**  **57/3**  **59/3**  **66/0**  **63/1** | **33.87**  **72.61**  **32.53**  **63.61**  **25.16** | **<0.0001**  **0.0859**  **<0.0001**  **<0.0001** |
|  | **WT**  ***dpy-10***  **FAT-7::GFP OE**  ***dpy-10* ; FAT-7::GFP OE**  ***dpy-10* ; *ptr-23* ; FAT-7::GFP OE** | **60/0**  **61/1**  **59/1**  **61/0**  **62/0** | **34.67**  **74.94**  **29.1**  **68.39**  **23.6** | **<0.0001**  **0.0011**  **<0.0001**  **<0.0001** |
|  | **WT**  ***dpy-10***  **FAT-7::GFP OE**  ***dpy-10* ; FAT-7::GFP OE**  ***dpy-10* ; *ptr-23* ; FAT-7::GFP OE**  ***ptr-23*** | **57/3**  **60/0**  **63/0**  **61/0**  **55/0**  **60/2** | **43.9.4**  **84.92**  **42**  **76.33**  **26.49**  **37.73** | **<0.0001**  **0.0351**  **<0.0001**  **<0.0001**  **0.0002** |
| **Heat Stress Survival @ 32˚C** | **WT ; 0mM Oleate**  **WT ; 0.8mM Oleate** | **57/4**  **59/1** | **39.29**  **31.47** | **<0.0001** |
|  | **WT ; 0mM Oleate**  **WT ; 0.8mM Oleate** | **59/1**  **58/0** | **30.19**  **25.69** | **<0.0001** |
|  | **WT ; 0mM Oleate**  **WT ; 0.8mM Oleate** | **61/0**  **61/0** | **32.1**  **36.33** | **<0.0001** |
| **Heat Stress Survival @ 32˚C** | **WT ; 0mM Linoleic acid**  **WT ; 0.8mM Linoleic acid** | **56/4**  **53/7** | **40.45**  **38.76** | **ns** |
|  | **WT ; 0mM Linoleic acid**  **WT ; 0.8mM Linoleic acid** | **57/3**  **60/0** | **70.11**  **58.61** | **ns** |
|  | **WT ; 0mM Linoleic acid**  **WT ; 0.8mM Linoleic acid** | **60/0**  **60/0** | **47.74**  **46.39** | **ns** |
| **Heat Stress Survival @ 32˚C** | ***dpy-10* ; 0mM Oleate**  ***dpy-10* ; 0.8mM Oleate** | **60/0**  **59/1** | **94.67**  **89.18** | **0.0318** |
|  | ***dpy-10* ; 0mM Oleate**  ***dpy-10* ; 0.8mM Oleate** | **60/0**  **60/0** | **102.25**  **64.17** | **0.0035** |
|  | ***dpy-10* ; 0mM Oleate**  ***dpy-10* ; 0.8mM Oleate** | **60/0**  **59/1** | **85.75**  **75.17** | **<0.0001** |
| **Heat Stress Survival @ 32˚C** | **WT**  ***fat-2*** | **52/8**  **52/8** | **53.67**  **36.67** | **<0.0001** |
|  | **WT**  ***fat-2*** | **59/1**  **60/0** | **57.78**  **34.22** | **<0.0001** |
|  | **WT**  ***fat-2*** | **60/0**  **60/0** | **39.83**  **28.70** | **<0.0001** |
|  | **WT**  ***fat-2*** | **58/2**  **58/2** | **46.11**  **28.72** | **<0.0001** |
| **Oxidative Stress Survival** | **WT ; 0mM Oleate**  **WT ; 0.8mM Oleate** | **60/0**  **60/0** | **5.09**  **5.07** | **ns** |
|  | **WT ; 0mM Oleate**  **WT ; 0.8mM Oleate** | **60/0**  **60/0** | **3.80**  **3.99** | **ns** |
|  | **WT ; 0mM Oleate**  **WT ; 0.8mM Oleate** | **60/0**  **60/0** | **3.28**  **3.49** | **0.0233** |
| **Osmotic Stress Survival**  **Osmotic Stress Survival** | **WT ; 0mM Oleate**  **WT ; 0.8mM Oleate** | **30/0**  **30/0** | **150.56s***  **148.83s*** | **ns** |
|  | **WT ; 0mM Oleate**  **WT ; 0.8mM Oleate** | **30/0**  **30/0** | **180.56s***  **169.69s*** | **ns** |
|  | **WT ; 0mM Oleate**  **WT ; 0.8mM Oleate** | **30/0**  **30/0** | **149.44s***  **149.22s*** | **ns** |

Statistics was calculated using the Kaplan Meier plot and Mantel-Cox test.
