# Supplementary material for: Homeostatic control of stearoyl desaturase expression via patched-like receptor PTR-23 ensures the survival of C. elegans during heat stress
Source: PLoS Genet. 2023 Dec 18;19(12):e1011067. doi: 10.1371/journal.pgen.1011067 (PMC10727360; doi:10.1371/journal.pgen.1011067)
Supplement: Attachment — Submitted filename: Response to Reviewers.pdf [file pgen.1011067.s008.pdf]

Reviewer #1: Venkatesh et al present their findings on the role of PTR-23 and certain fatty acids, specifically oleate, in the resistance to heat stress. They propose a model where PTR-23 inhibits the expression of the fatty acid desaturase fat-7, which decreases the amount of monounsaturated oleic acid, promoting animal survival during exposure to high temperature. These findings are of interest as they demonstrate a non-canonical role for the hedgehog signaling pathway, or at least it's repurposing by C. elegans. The manuscript is well-written and easy to follow.

Response: We would like to thank the reviewer for their valuable comments/suggestions. Please note that the line numbers have changed in the revised manuscript due to edits.

I only have a few small suggestions / comments:
ptr-23(RNAi) worms are more sensitive to heat shock. Is this specific? I.e. what about hyperosmotic stress that was used as a non-specific control for fat-7 mutants or fatty acid supplementation?

Response: *ptr-23* RNAi had WT phenotype upon hyperosmotic stress and oxidative stress (results presented in Fig. S5, B-C).

How does PTR-23 inhibit expression of fat-7? I understand that addressing this experimentally would be beyond the scope of the manuscript, but can the authors add to the discussion section their ideas for the signal transduction pathway, particularly given that C. elegans does not have full hedgehog signaling pathway present.

Response: There are several possibilities. PTR-23 might regulate FAT-7 expression through known TFs such as SBP-1, MDT-15, NHR-49, MHL-3, HLH-30, NHR-64, and NHR-80 (Lee *et al.*, 2015; Van Gilst *et al.*, 2005; O'Rourke *et* al., 2013; Liang *et* al., 2010; Brock *et al.*, 2006). Since PTR-23 appears to function in the hypodermis and FAT-7 in the hypodermis and the intestine, a cell non-autonomous means of FAT-7 regulation is warranted. Some of this is included in the Discussion section of the revised manuscript.

Labels in Figure 1C are too small. For the pathway diagram to be useful, we should be able to see and read what is there.

Response: We have enlarged Figure 1C in the revised manuscript. Thanks for this suggestion.

Are the GC-MS data for WT at 20C in Fig 1C and Fig 3A actually exactly same data? Were all the samples for the MS collected at the same time, in parallel?

Response: The WT GC-MS data at 20˚C is the same in Fig. 1 and Fig. 3, however, they are compared with different genotypes in these two figures. We have mentioned this in the legend for Figure 3. For each genotype and heat shock treatment, 7 to 18 biological replicates were collected over two different days. Combined data of all sets are presented.

Do not use strain names (e.g. NR222) in legends. It makes it harder to understand what is going on. Instead use gene names.

Response: We understand that strain names will be confusing for unfamiliar readers. We have added respective genotypes in the legend.

While overall the manuscript is well-written, there are some typos that need to be taken care of. For example, the first sentence of the abstract: “Organismal responses to temperature fluctuations includes an evolutionary conserved cytosolic chaperone machinery as well as…” Should be corrected to “responses … include” (i.e., correct agreement in plurality).

Response: Thank you for pointing such typos. We have proofread the manuscript and revised any typos and grammatical errors.

Reviewer #2: Venkatesh et al. PLoS Genetics 2023

In this work, Venkatesh et al. demonstrate that the spectrum of saturated and unsaturated fatty acids changes in heat stress animals. They demonstrate that the MUFA oleic acid and the enzyme FAT-7 that synthesizes it reduce heat resistance. They also analyze links between fatty acid desaturases and heat stress in permeability-defective dpy-10 mutants and mutants in Patched receptor-related PTR-23. The experiments convincingly show coordination between fatty acid desaturation and heat stress resistance. The conclusions about the relationship of PTR-23 to these components are less well supported.

Response: We would like to thank the reviewer for their valuable comments/suggestions.

Major comments

1. Double mutant analyses presented suggest that PTR-23 and DPY-10 act independently to regulate heat stress response, because the heat resistance of dpy-10;ptr-23 is intermediate between those of dpy-10 and ptr-23, rather than demonstrating epistasis. The best evidence that ptr-23 acts downstream of dpy-10 is the regulation of ptr-23 transcripts in the dpy-10 mutant. The two conclusions could be reconciled by proposing other target genes downstream of dpy-10 that also contribute to its phenotype.

Response: We agree with the reviewer's view. *dpy-10* animals have elevated transcript levels of *ptr*-23 (this study and 2010 paper Lametina) which drive some of the response we have described in this study. However, *dpy-10* may have additional mechanisms turned on such as those regulating stress response, such as glutathione S transferases (Sandhu, 2021) and chaperones such as small heat shock response proteins (data not shown).

2. Figure 5 is missing the panel with lipid analysis after heat shock.

Response: Lipid analysis after heat shock is provided in full in Supplementary Table S1. In Fig 5D, we wished to focus on differences that exist at the basal level.

3. The authors should consider the possibility that PTR-23 acts as a lipid transporter rather than as a receptor, an interpretation that has been presented in recent papers. An issue with the receptor model is that a receptor should act cell autonomously in the tissue in which it is expressed, but this study demonstrates that it does not.

Response: This idea is indeed possible. In this study, we show that PTR-23 regulates FAT-7 expression in the *dpy-10* animals in heat-stressed WT animals. Through tissue-specific RNAi, we show that *ptr-23* functions in the worm's cuticle to regulate FAT-7 levels However, from the FAT-7::GFP lines, it is clear that maximal expression of FAT-7 is in the intestine. Therefore, we do not have any strong evidence to suggest that cuticular *ptr-23* might transport OA in the intestine, nor is there a clear link for how such transport might affect transcript level. Moreover, regulation of a gene at the transcript and protein levels is a hallmark of signalling cascades. For this reason, we have refrained from discussing the potential transport of lipids by PTR-23.

Minor comments/typos
Title: I'm not sure that "Homeostatic" is the best description for this type of control, because it implies maintenance of a condition, rather than a stress response.

Response: We have used "homeostatic" term to highlight the maintenance role played by PTR-23 in regulating FAT-7 and OA levels in the WT animals experiencing physiologically relevant scenarios, such as heat stress or skin permeability due to *dpy-10* mutation.

Abstract: "includes an evolutionary" should be "include an evolutionarily"

Response: Thank you for pointing this out. We have corrected this. Further, we have proofread the manuscript and have corrected any typos and grammatical errors.

What is meant by "physiologically relevant" heat stress? Are these conditions that would be expected in the natural habitat?

Response: *C. elegans* is often found in decaying vegetation and compost. In compost heaps temperatures can easily reach above 40C (https://compost.css.cornell.edu/physics.html). Therefore, the 32˚C stress we have used in this study is likely to be physiologically relevant stress.

Fig. 1B How long is the heat stress condition that was applied in this experiment (fat gene RT-PCR)?

Response: The worms were subjected to heat stress for 8 hours. We have also included data on levels of FAT genes upon heat stress of 4, 8 and 12 hours in Fig. S1.

Fig. 2E, F are not discussed in the text

Response: Thanks for pointing this out. We have described it in the revised manuscript.

p. 10-11 The authors show that heat stress causes permeability defects in the cuticle. This result is presented as supporting the conclusion that permeability defective dpy-10 mutants cause defects in heat stress, but I find that it puts the direction of causality into question. Because of this circular causation, a simpler explanation is that permeability and heat stress response are jointly regulated by something else. Can the authors discuss their ideas for how/why heat stress causes permeability defects in the context of their model?

Response: There is evidence that permeability determining collagen levels are affected by different stresses. For example, *dpy-10* transcripts are upregulated when worms are treated with oxidative stress-inducing agent, paraquat (Sandhu, 2021). It is possible that cuticular collagens experience denaturation during heat stress. Derman et al have shown that collagens are indeed affected by temperature (10.1007/s12195-020-00653-w).

p. 11 "no effect of PTR-23 on other desaturases" According to the supplemental data, there are significant differences in expression of the other desaturases.

Response: We have revised the manuscript to state that PTR-23 regulates expression of other fatty acid desaturases.

p. 11 “cutilcle” should be “cuticle”

Response: Thanks for pointing this out. We have corrected this.

p. 14 “suppression of FAT-7 expression” would be more accurately worded “repression of FAT-7 expression”

Response: The suggested change has been made in the revised manuscript.

p. 14 “To study the impact of PTR-23 on oleic acid levels, we studied the levels of fat-7” is not consistent. Do the authors mean “To study how PTR-23 impacts oleic acid levels”?

Response: This was the intended meaning. We have corrected this statement for clarity.

Fig. 6 title “PTR-23 regulates fatty acid levels” does not describe the experiments shown.

Response: The title of Fig. 6 has been changed to “PTR-23 regulates stearoyl desaturase FAT-7 levels in *dpy-10.*”

Fig. 6 – It would be helpful to have statistics that compare heat shock to control for each genotype (rather than comparisons of genotypes under the same conditions).

Response: The purpose of this figure was to highlight that *ptr-23* is essential for downregulating *fat-7* transcripts and GFP-fusion proteins in *dpy-10* animals. For this reason, genotypes were compared rather than the conditions.

Reviewer #3: The study by Venkatesh, Siddiqui, Sandhu, and colleagues investigated how lipid desaturation contributes to the response of C. elegans to heat stress. Specifically, the study focused on whether unsaturated fatty acids, like oleic acid, negatively affect survival during prolonged stress. The authors find that decreasing the levels of polyunsaturated fatty acids by suppressing desaturase enzymes plays a crucial role in the worms' response to heat stress. Accordingly, excessive oleic acid is harmful to survival under chronic heat stress conditions.
The study also explored the connection between cuticle collagen defects and lipid balance within the worm's body. In heat-stress-resistant dpy-10 worms, the expression of fat-7 is decreased, leading to a decrease in unsaturated fatty acids and an increase in saturated fatty acids. The authors further discovered that the heat stress resistance of the dpy-10 mutant is regulated by the epidermal patched-like receptor PTR-23, which serves as a negative regulator of fat-7 expression.

The topic of this paper is of great and broad interest. In particular this study provides new insight into the role of an epidermal patched-like receptor and thus of a C. elegans epidermis to intestine axis in heat stress adaptation. The presented results are mostly (see my comments below) convincing and often validated by different experimental approaches (e.g. using single and double mutants and tissue-specific RNAi for functional analyses, supplementation experiments for determining the role of oleic acid, or gas chromatography-mass spectrometry for assessing fatty acid composition in various genetic backgrounds). My main reservation concerns the presentation of the survival data, since it does not become clear if the graphs that are shown are based on data from multiple or single experimental runs and if any statistical analysis was done to determine P-values . I found the survival statistics in Table S2 eventually, but I strongly recommend to present the data in a way that the reader can understand everything from the figure legend (also see my comments below).

Response: Thanks for the comments. Each of these graphs are representative and each of these survival experiments were performed at least three times. Mantel-Cox (log-rank) test was performed on all curves and $p < 0.05$ was considered significant. Table S2 presents all the statistics pertaining to the survival experiments in a standard form. Most studies that include survival curves represent the corresponding statistics in this standardized form (Sandhu *et al*., 2021; Dasgupta *et al*., 2020; Alcedo & Kenyon, 2004). Additional description is provided in Figure legends and in Table S2 in the revised manuscript.

Minor comments:
line 88: delete ‘by’

Response: We have made this change.

line 128/129: I suggest to refer to Fig 1C in line 125-126 (and omit this sentence)

Response: Thank you for this suggestion. The necessary edits have been made.

line 136: Please mention the gene name (dgat-2)

Response: Gene name mentioned in the revised manuscript.

line 141: What is meant by ‘exquisitely’ in this context?

Response: We have changed it to ‘intricately, to convey complex regulation.

line 213: Please replace ‘are linked’ by ‘is linked’

Response: This change has been made. Thank you.

line 216/217: Was this an unexpected finding? Since DPY-10 is a permeability-determining collagen and heat-stress causes permeability defects, it is not obvious why mutations in dpy-10 lead to enhanced survival during heat stress.

Response: Our study reveals that either heat stress or permeability defect (due to dpy-10 mutation) lead to the upregulation of PTR-23. Dpy-10 has enhanced levels of ptr-23 at the basal level (consequently reduced levels of FAT-7 and OA) protecting them from heat stress. HSR of dpy-10 mutants due to altered MUFA levels (than altered chaperones) was indeed an unexpected finding.

Survival data in e.g. Fig 2 A-D, Figure 4 C, E, and F, Figure S5 A-D: Please add sample size (i.e. 25-30 worms according to methods section) and number of experimental runs to figure legend. Do the Kaplan-Meier survival curves show a representative run or pooled data? How many experimental runs were done? Where do I find the raw data? The graphs show the Kaplan-Meier survival curves, but was any statistical analysis done to determine P-values?

Response: Thanks for these suggestions. The necessary n and N values have been included. The Kaplan-Maier curves shown here are all representative. Each experiment was performed at least thrice, and the source data and statistics are described in Table S2. p values were determined using Mantel-Cox log-rank test.

Fig 4: In the legend, please explain the NR222 strain, so that the reader does not have to look into the main text to understand what was done.

Response: We understand that strains names will be confusing for unfamiliar readers. We have replaced strain names in the legends with their genotype.

Fig 7: The worm looks rather like an earthworm.

Response: The rings represent the uniform arrangement of the annular rings in the cuticle, which is disrupted in *dpy-10* mutants (Sandhu 2021, Essmann 2021).

REFERENCES:

Sandhu A, Badal D, Sheokand R, Tyagi S, Singh V. Specific collagens maintain the cuticle permeability barrier in Caenorhabditis elegans. Genetics. 2021 Mar 31;217(3):iyaa047. doi: 10.1093/genetics/iyaa047. PMID: 33789349; PMCID: PMC8045729.

Dasgupta M, Shashikanth M, Gupta A, Sandhu A, De A, Javed S, Singh V. NHR-49 Transcription Factor Regulates Immunometabolic Response and Survival of Caenorhabditis elegans during Enterococcus faecalis Infection. Infect Immun. 2020 Jul 21;88(8):e00130-20. doi: 10.1128/IAI.00130-20. PMID: 32482643; PMCID: PMC7375755.

Alcedo J, Kenyon C. Regulation of C. elegans longevity by specific gustatory and olfactory neurons. Neuron. 2004 Jan 8;41(1):45-55. doi: 10.1016/s0896-6273(03)00816-x. PMID: 14715134.

Lee D, Jeong DE, Son HG, Yamaoka Y, Kim H, Seo K, Khan AA, Roh TY, Moon DW, Lee Y, Lee SJ. SREBP and MDT-15 protect C. elegans from glucose-induced accelerated aging by preventing accumulation of saturated fat. Genes Dev. 2015 Dec 1;29(23):2490-503. doi: 10.1101/gad.266304.115. PMID: 26637528; PMCID: PMC4691952.

Van Gilst MR, Hadjivassiliou H, Jolly A, Yamamoto KR. Nuclear hormone receptor NHR-49 controls fat consumption and fatty acid composition in C. elegans. PLoS Biol. 2005 Feb;3(2):e53. doi: 10.1371/journal.pbio.0030053. Epub 2005 Feb 8. PMID: 15719061; PMCID: PMC547972.

Brock TJ, Browse J, Watts JL. Genetic regulation of unsaturated fatty acid composition in C. elegans. PLoS Genet. 2006 Jul;2(7):e108. doi: 10.1371/journal.pgen.0020108. Epub 2006 Jun 5. PMID: 16839188; PMCID: PMC1500810.

O'Rourke EJ, Ruvkun G. MXL-3 and HLH-30 transcriptionally link lipolysis and autophagy to nutrient availability. Nat Cell Biol. 2013 Jun;15(6):668-76. doi: 10.1038/ncb2741. Epub 2013 Apr 21. Erratum in: Nat Cell Biol. 2015 Jan;17(1):104. PMID: 23604316; PMCID: PMC3723461.

Liang B, Ferguson K, Kadyk L, Watts JL. The role of nuclear receptor NHR-64 in fat storage regulation in Caenorhabditis elegans. PLoS One. 2010 Mar 25;5(3):e9869. doi: 10.1371/journal.pone.0009869. PMID: 20360843; PMCID: PMC2845610.

Clara L. Essmann, Muna Elmi, Mike Shaw, Giridhar M. Anand, Vijay M. Pawar, Mandayam A. Srinivasan. In-vivo high resolution AFM topographic imaging of Caenorhabditis elegans reveals previously unreported surface structures of cuticle mutants. Nanomedicine: Nanotechnology, Biology and Medicine, Volume 13, Issue 1, 2017, Pages 183-189, https://doi.org/10.1016/j.nano.2016.09.006.

-----------------------------------End of Response---------------------------------------
